# Appropriate Macronutrients or Mineral Elements Are Beneficial to Improve Depression and Reduce the Risk of Depression

**DOI:** 10.3390/ijms24087098

**Published:** 2023-04-12

**Authors:** Zhengyang Quan, Hui Li, Zhenzhen Quan, Hong Qing

**Affiliations:** Key Laboratory of Molecular Medicine and Biotherapy, School of Life Science, Beijing Institute of Technology, Beijing 100081, China

**Keywords:** depression, macronutrients, mineral elements, appropriate supplementation, overdose or deficiency

## Abstract

Depression is a common mental disorder that seriously affects the quality of life and leads to an increasing global suicide rate. Macro, micro, and trace elements are the main components that maintain normal physiological functions of the brain. Depression is manifested in abnormal brain functions, which are considered to be tightly related to the imbalance of elements. Elements associated with depression include glucose, fatty acids, amino acids, and mineral elements such as lithium, zinc, magnesium, copper, iron, and selenium. To explore the relationship between these elements and depression, the main literature in the last decade was mainly searched and summarized on PubMed, Google Scholar, Scopus, Web of Science, and other electronic databases with the keywords “depression, sugar, fat, protein, lithium, zinc, magnesium, copper, iron, and selenium”. These elements aggravate or alleviate depression by regulating a series of physiological processes, including the transmission of neural signals, inflammation, oxidative stress, neurogenesis, and synaptic plasticity, which thus affect the expression or activity of physiological components such as neurotransmitters, neurotrophic factors, receptors, cytokines, and ion-binding proteins in the body. For example, excessive fat intake can lead to depression, with possible mechanisms including inflammation, increased oxidative stress, reduced synaptic plasticity, and decreased expression of 5-Hydroxytryptamine (5-HT), Brain Derived Neurotrophic Factor (BDNF), Postsynaptic density protein 95(PSD-95), etc. Supplementing mineral elements, such as selenium, zinc, magnesium, or lithium as a psychotropic medication is mostly used as an auxiliary method to improve depression with other antidepressants. In general, appropriate nutritional elements are essential to treat depression and prevent the risk of depression.

## 1. Introduction

Depression is one of the most common mental disorders globally, with an estimated 280 million people in the world suffering from it [1]. At worst, severe depression can lead to suicide. Not only does depression bring mental problems to the patients themselves, but it also causes financial and social burdens to their families and society [2].

The monoamine theory has influenced the development of major antidepressant treatments, including monoamine oxidase inhibitors (MAOIs), selective serotonin reuptake inhibitors (SSRIs), and serotonin-norepinephrine reuptake inhibitors (SNRIs). These inhibitors are functional by increasing monoamine levels (5-hydroxytryptamine, norepinephrine) to treat depression [3]. With accumulated studies on depression, other biochemical and physiological factors have also been implicated in the pathogenesis of depression, including brain-derived neurotrophic factor (BDNF)-related neurotrophic atrophy [4], inflammation [5], hypothalamic–pituitary–adrenal (HPA) axis dysfunction [6], etc. Among them, the role of nutrients in depression has attracted more and more attention [7].

**Figure 1:**
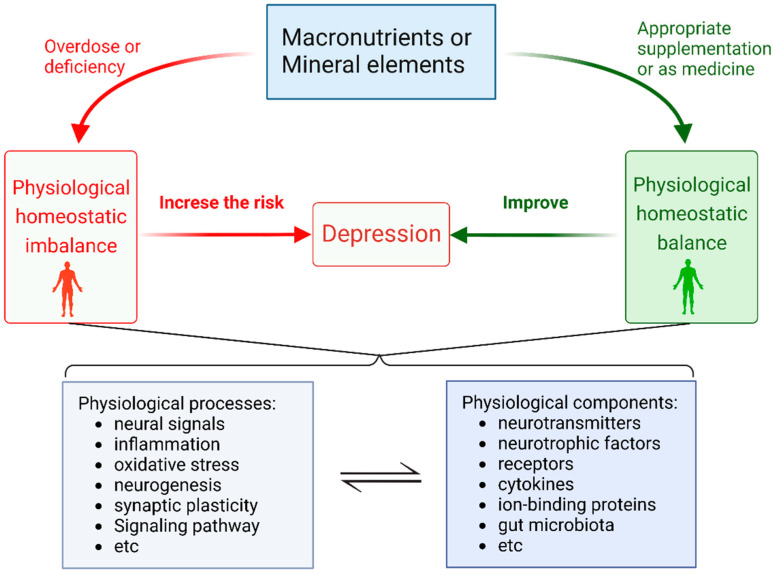
Illustrating the relationship between depression and macronutrients or mineral elements and possible mechanisms including physiological processes and components.

The nutritional elements have been reported to help maintain a stable mental state, and an imbalance of them is closely related to depression [8]. As shown in Figure 1, this review will explore the relationship between the imbalance of some nutrients and depression and summarize that the excess or deficiency of nutrients can increase the incidence of depression, thus maintaining the balance of corresponding nutrients can help reduce the incidence of depression. Moreover, appropriate supplementation of some mineral elements is considered to help treat depressed patients. The possible physiological processes and molecular mechanisms involved are discussed based on experiments.

## 2. Overdose or Deficiency of Macronutrients Elements Increase the Risk of Depression

### 2.1. Dietary Sugars

Glucose is the primary source of energy for the human brain. ATP produced by glucose metabolism is the basis for maintaining neuronal and non-neuronal cell functions in the brain, such as producing neurotransmitters and nerve impulses [9]. Most sugars are metabolized in the body to produce glucose. There are many sugars in sweets, beverages, and candies. Many studies have shown that the excessive intake of sweets, sugar-sweetened beverages, and candy increases the risk of depression. Guo et al. have shown that regular consumption of sugar-sweetened beverages might increase the risk of depression in older Americans [10]. A study by Vermeulen et al. in a Dutch population also showed that a dietary pattern high in sugar (HS) increases the risk of depression [11]. The result of a 3-year follow-up survey by Shimmura et al. showed that high candy consumption significantly increases the risk of depression among Japanese workers, with 16.8% of high candy eaters experiencing depressive symptoms [12]. A study by Kashino et al. also showed that Japanese people who drink ≥4 cups of sugar-sweetened beverages per week have a 91% higher risk of depression than those who drink <1 cup/week [13]. A meta-analysis study indicated that people who consume 2 cups of cola per day have a 5% increased risk of depression, while those who consume the equivalent of 3 cans of cola per day have an approximately 25% increased risk of depression [14]. The research among Chinese people has also demonstrated that a high-sugar diet increases the odds of depression [15,16]. A study in the Spanish population found that consumption of added sugars was associated with a significantly increased risk of depression but no significant association between the consumption of sugar-sweetened beverages and the risk of depression [17]. A study on the Korean population suggested that beverage intake increases the risk of depression in women but decreases the risk in men. The differences may be due to different statistical methods for sugar intake and evaluation criteria for depression [18]. Moreover, a high-sugar diet is prone to diabetes and obesity, which are also risk factors for depression [19,20]. In addition to sugar, sugary drinks and desserts may add sweeteners and other ingredients, the excessive intake of which may also be associated with the occurrence of depression, but there is currently a lack of relevant research with follow-up studies. A study has shown that fasting blood glucose concentrations (FBG) were significantly elevated in major depressed patients compared to healthy subjects (4.73 ± 0.45 vs. 4.52 ± 0.43 mmol/L, *p* < 0.01) [21].

The possible physiological processes and physiological components of a high-sugar diet affecting depression might be considered in the following pathways: 1. Neural signals: it affects the content of 5-Hydroxytryptamine (5-HT) in the brain. Animal experiments showed that a high-sugar diet reduces the activity of dendritic 5-HT-1A receptors, which may impede the feedback control of serotonin synthesis and release in the hypothalamus leading to a decrease in 5-HT [22]. 5-HT is a crucial monoamine neurotransmitter, and its decreased content in the brain is one of the critical factors leading to depression [23]. 2. Inflammation and pro-inflammatory factors. A meta-analysis study by Köhler et al. indicated that pro-inflammatory factors such as interleukin-6(IL-6), tumor necrosis factor-α(TNF-α), interleukin-13(IL-13), interleukin-12(IL-12), etc., are significantly elevated in major depressive disorder (MDD) patients, which associates inflammation with depression [24]. Lipopolysaccharide (LPS) is a commonly used inflammatory inducer. Experimental studies have shown that LPS induces inflammation in rodents at the same time as depression-like symptoms [25,26], indicating there might be a correlation between inflammation and depression. Do et al. showed that a high-sugar diet could induce inflammation and depression-like behavior in mice. Moreover, they found that a high-sugar diet may induce inflammation by altering the gut microbiota and intestinal permeability [27]. 3. Synaptic plasticity and the expression of brain-derived neurotrophic factor (BDNF). The level of BDNF in the serum of patients with MDD is significantly lower than that of healthy patients, and after receiving antidepressant treatment, the level of BDNF in the patient’s body is significantly increased. BDNF can be used as a biomarker of depression or as a measure of antidepressant efficacy predictors [28]. Another study showed that low plasma BDNF is associated with suicidal behavior in major depression [29]. BDNF is widely expressed in the developing and adult mammalian brain and has been implicated in development, neural regeneration, synaptic transmission, synaptic plasticity, and neurogenesis [30]. A deficiency of BDNF or Trk receptors does not induce depression, but antidepressants are required to increase BDNF activity and restore neuronal networks [31]. In rodent models, a high-glucose diet can reduce the expression of BDNF, synapsin I, cyclic AMP-responsive element-binding protein (CREB), and growth-associated protein 43, which affect synaptic plasticity [32]. Another study showed that after one week of feeding rats with high sugar and fat, dendritic spines and dendritic branches in the CA1 region of the rat brain were significantly reduced [33].

### 2.2. Dietary Fat

A study showed that fat content is a risk factor for depression [34]. Fat accumulation in the body leads to obesity, which is also a risk factor for depression. A meta-analysis displayed that obese individuals have an 18% increased risk of depression [35]. An intracerebral study showed a 40% increased risk of depression in obese adolescents [36]. After dietary fat is metabolized and absorbed by the human body, it will be mainly converted into triglycerides (TG), total cholesterol (TC), etc. High-density lipoprotein cholesterol (HDL-C) and low-density lipoprotein cholesterol (LDL-C) are the main components of total cholesterol. TG, TC, HDL-C, and LDL-C are four items of blood lipid tests [37]. Peng et al. showed that HDL-C in the blood is significantly higher in major depressed patients compared to healthy subjects (1.31 ± 0.32 vs. 1.24 ± 0.300 mmol/L, *p* < 0.01); however, there are no significant changes in LDL-C, TC, and TG [21]. Another study showed a significant association between high levels of HDL-C (≥1.04 mmol/L) and depression in adult men and between high levels of TG (≥1.7 mmol/L) and depression in adult women [38]. However, Enko et al. observed that HDL-C is significantly lower in major depressed patients compared to healthy subjects (1.43 [1.97–4.01] vs. 1.60 [1.23–1.89] mmol/L, *p* = 0.049), and TG is significantly higher (1.08 [0.76–1.54] vs. 0.84 [0.63–1.32] g/L, *p* = 0.014) [39]. A recent Mendelian randomization analysis by So et al. reported a positive association of HDL-C with major depressed patients, but increased HDL-C is causally associated with fewer depressive symptoms. The reasons for the discrepancy may involve the different evaluation criteria for depression and the heterogeneity of samples [40]. It suggests that abnormal HDL-C and TG may be risk factors for depression, which need further research. In addition to research in humans, there is a similar phenomenon in rodents. Mice given a high-fat diet (HFD) for 12 weeks developed depressive-like behaviors, and then switching the high-fat diet to a standard diet for 4 weeks eliminated the depressive-like behaviors in mice [41]. After administration of an HFD in BALB/c mice, high-density lipoprotein cholesterol and low-density lipoprotein cholesterol are strongly associated with depressive-like behavior [42]. The study by Anders et al. showed HFD could exacerbate depressive-like behaviors in the Flinders Sensitive Line (FSL) rat [43]. Another study also suggested that olive leaf extract may prevent depression by inhibiting fat mass and weight gain in mice fed with a high-fat diet [44].

The possible physiological processes and mechanisms of a high-fat diet affecting depression are summarized as follows: 1. Neural signals: 5-HT, glutamatergic receptor, GABA_A_ receptor, glutamate, and aspartate transporter. After feeding with HFD for 14 weeks, Wu et al. found a significant decrease in the 5-HT system expression in the hippocampus of C57BL/6 mice [45]. A study also showed that HFD attenuated the inhibitory effect of escitalopram, a selective serotonin reuptake inhibitor (SSRI), on 5-HT reabsorption in the brain, reducing the concentration of 5-HT in synapses [46]. A high-fat diet administration of intestinal 5-HT synthesis inhibitors can attenuate depression-like behaviors in mice with high-fat diet-induced depression [47]. Long-term use of HFD can induce depressive-like behavior in rats and lead to decreased expression levels of the AMPA receptor (GlutA2) and GABA receptor (GAD65) [48]. HFD-induced depression correlates with the desensitization of GABAergic AgRP (agouti-related peptide) neurons in the hypothalamus, which plays a fundamental role in the control of appetite and body weight [49]. A recent study suggested that feeding mice an HFD causes the downregulation of glutamate transporter 1 (GLT-1), leading to glutamate overactivation, which in turn leads to depression [50]. 2. Inflammation and oxidative stress. A high-fat diet can induce an increase in proinflammatory cytokines in the rat hippocampus and depression-like behaviors [46]. In HFD-fed rats, depressive-like behaviors develop due to the overproduction of proinflammatory cytokines TNF-tumor necrosis factor alpha (TNF-α), interleukin-6 (IL-6), and interleukin-1 beta (IL-1β), the oxidative stress-related elevation of thiobarbituric acid-responsive substances (TABRS), and the down-regulation of antioxidant enzymes catalase (CAT) and glutathione peroxidase (GPX). Antidepressant agomelatine (AGO) eliminated depression in HFD rats, reduced the activity of inflammatory cytokines (TNF-α, IL-6, and IL-1β), TABRS, and restored the activity of CAT and GPX [51]. The antidepressant simvastatin (SMV) might also ameliorate depression by reducing inflammation in the brains of HFD-fed mice [45,52]. 3. Synaptic plasticity. Studies have shown that HFD also affects synaptic plasticity by reducing the expression of βIII-tubulin, postsynaptic density protein 95(PSD-95), synaptosomal-associated Protein, 25 kDa (SNAP-25), and neurotrophic factor-3 when it causes depression-like behavior in rats [48]. 4. The involvement of signaling pathways. HFD may induce depression in rats by desensitizing the Akt/GSK3β signaling pathway to 5-HT in the DG subgranular region of the hippocampal dentate gyrus, and returning to a normal diet can rescue the Akt/GSK3β response to 5-HT and alleviate depression-like behaviors [53]. Mice exposed to an HFD show accumulated fatty acids in the hypothalamus, leading to depression by inhibiting the cAMP/PKA signaling cascade [54]. HFD might also inhibit AMPK phosphorylation and induce mTOR phosphorylation to suppress autophagy, thus leading to depression-like behavior in mice [55]. 5. Other related receptor proteins: leptin receptor long isoform (LepRb), cannabinoid receptor type 1 (CNR1). LepRb plays an important role in regulating depression and anxiety-related behaviors, and selective deletion induces depression-related behaviors [56,57]. Yang et al. showed that high fat can cause depressive-like behaviors in rats and result in reduced levels of LepRb protein and mRNA in the hippocampus and hypothalamus [58]. CNR1, an important component of the endocannabinoid system, plays an important role in depression [59]. CNR1-deficient mice can also be used to model depression in mice [60]. A study showed that pregnant rats fed an HFD led to depressive-like behaviors in their offspring, with a decrease in the *Cnr1* mRNA levels in the prefrontal cortex in the male offspring [61].

### 2.3. Dietary Protein

There are fewer studies on the relationship between dietary protein and depression. Low-protein diets are associated with an increased risk of depression in the U.S. and Korean populations. Among macronutrients carbohydrates, protein, and fat, the prevalence of depression decreases significantly in both the United States and South Korea when the proportion of calories consumed from protein increases by 10% [62]. Another study in the United States showed that an increase in protein intake reduces the risk of depression in men but increases the risk of depression in women [63]. A cross-sectional study suggested that total protein intake from milk and dairy products may reduce the risk of depressive symptoms in U.S. adults [64]. In a population of Japanese male workers, a study suggested that low protein intake may be associated with a higher prevalence of depressive symptoms [65]. Peng et al. showed that total protein (TP) is significantly decreased in major depressed patients compared to healthy subjects (4.73 ± 0.45 vs. 4.52 ± 0.43 mmol/L, *p* < 0.01) [21]. Red and processed meats contain protein and saturated fat, and excessive consumption of either could slightly increase the risk of depression [66]. Low protein intake reduces depressive symptoms in diabetic patients [67]. Milk is also rich in protein and fat, and intake of skim milk is inversely associated with depression, while whole milk is positively associated with depression [68].

Dietary protein is rich in amino acids, which can supplement the amino acids required by the human body to maintain normal physiological functions. Tryptophan in dietary protein is a precursor for the synthesis of serotonin, and an increase in serotonin in the brain is the key to treating depression [69]. A survey by Euter et al. found that a diet low in tryptophan is associated with a higher risk of depression [70]. Subchronic tryptophan depletion is also used as an animal model of depression [71]. Tryptophan in dietary protein is also a precursor compound for synthesizing dopamine, which has also been implicated in antidepressant therapy [72]. In milk proteins, alpha-lactalbumin [73] and lactoferrin [74] also help improve depression-like symptoms in mice.

## 3. Overdose or Deficiency of Mineral Element Increase the Risk in Depression

### 3.1. Zinc(Zn)

Zinc is an essential trace element that plays an important role in many biochemical and physiological processes in relation to brain growth and function [75]. Studies in many national populations, such as the United States [76], Australia [77], and Japan [78,79,80], found that a lack of dietary zinc intake increases the risk of depression. Two other studies have shown that insufficient dietary zinc intake leads to depressive symptoms in women but not in men [81,82]. Al-Fartusie et al. showed that zinc in serum is significantly lower in major depressed patients compared to healthy subjects (0.72 ± 0.08 vs. 0.96 ± 0.11 mg/L, *p* < 0.01) [83]. Islam et al. found the same experimental results [84]. In rodents, a zinc-deficient diet also induced depressive-like behavior [85,86,87].

We also summarized the possible physiological processes and mechanisms of zinc in depression. 1. It is related to zinc transporters (ZnTs). In mammals, zinc homeostasis is primarily regulated by ZnTs [88]. A study showed that there are significant increases in protein levels of ZnT1, ZnT4, and ZnT5 in the prefrontal cortex in MDD but a reduced protein level of ZnT3 [89]. Zinc transporter 3 (ZnT3) plays an important role in concentrating zinc ions within synaptic vesicles in a subset of the brain’s glutamatergic neurons [90]. In the stress-induced rat depression model, total zinc levels were reduced, and the mRNA expression of *ZnT1* and *ZnT3* was significantly reduced in the hippocampus [91]. Neurogenesis in the hippocampus was reduced in both rats fed with a zinc-deficient diet and *ZnT3* knockout mice, but it was resumed after a normal zinc diet treatment [92]. 2. It is related to Zn^2+^-activated G protein-coupled receptor 39 (GPR39). GPR39 senses changes in extracellular zinc concentrations, which results in the activation of an intracellular signaling pathway to regulate the expression of genes associated with depression, such as BNDF and 5-HT [93,94]. GPR39 knockout causes depressive-like behavior in mice [95]. Depressive-like symptoms were observed in *GPR39* knockout mice, accompanied by decreased *CREB* and *BDNF* expression [96]. The GPR39 protein can bind to 5-HT1A and form a 5-HT1A-GPR39 complex that is regulated by zinc concentration [97]. 3. Inflammation and oxidative stress. After giving rats a zinc-deficient diet for 6 weeks, Doboszewska et al. found that it causes depressive behavior and increases the oxidation/inflammation parameters IL-1 and TBARS in rats [98]. 4. N-methyl-d-aspartate (NMDA). NMDA has emerged as a therapeutic target for depression therapy in clinical and preclinical studies. Since increasing evidence has supported the disruption of glutamate homeostasis and neurotransmission in depressed subjects [99]. A study has shown that in rats, zinc deficiency-induced depression-like behaviors are associated with increased NMDAR (GluN1, GluN2A, GluN2B), decreased AMPAR(GluA1), p-CREB, and BDNF in the hippocampus to change the NMDAR neuronal signal [100]. Another study also showed that in rats, zinc deficiency-induced depression-like behaviors are associated with increased NMDAR (GluN2A and GluN2B), decreased PSD-95, p-CREB, and BDNF in the hippocampus [101].

### 3.2. Magnesium (Mg)

Magnesium is one of the most important minerals in the human body and is involved in various biological processes in the brain and the fluidity of neuronal membranes, maintaining the stability of brain function [102]. Multiple studies have shown that dietary magnesium intake is inversely associated with the risk of depression [103,104,105]. Moreover, magnesium in serum is significantly lower in major depressed patients compared to healthy subjects (1.10 ± 0.11 vs. 1.64 ± 0.15 mg/L, *p* < 0.01) [79]. Like humans, a magnesium-deficient diet induces rodent depression-like behaviors [106,107].

The possible regulatory mechanisms of magnesium in depression could involve gut microbiota, NMDA nerve signaling, and oxidative stress: Magnesium deficiency diet might lead to depression-like behavior possibly by altering intestinal microbiome composition and inducing homeostasis of the microbiome–gut–brain axis in mice [107]. Another study showed that dietary Mg supplementation increases bacteria involved in intestinal health and metabolic homeostasis and reduces bacteria involved in inflammation and human diseases [108]. Ghafari et al. showed that enhancement of depressive-like behaviors induced by dietary magnesium restriction is associated with decreased levels of amygdala-hypothalamic proteins of GluN1-containing NMDA complexes [109]. Whittle et al. showed that mice fed a low Mg-containing diet (10% of the daily requirement) exhibit depression-like behavior and elevated expression of N(G), N(G)-dimethylarginine dimethylaminohydrolase 1 (DDAH1), manganese-superoxide dismutase (MnSOD), and glutamate dehydrogenase 1 (GDH1) related to oxidative stress [110]. Another study also showed that depression is associated with a decrease in magnesium concentrations in the human body, which leads to an increase in GPX associated with oxidative stress [111].

### 3.3. Copper(Cu)

Copper is an important trace element required by essential enzymes. However, copper also leads to the production of toxic reactive oxygen species due to its redox activity, so copper uptake is strictly controlled [112]. It was reported that the serum of major patients with depression contains higher levels of copper compared to healthy subjects (1.55 ± 0.12 vs. 1.12 ± 0.13 mg/L, *p* < 0.01) [79]. And the same results were given by the Islam team [84] and the Ni team [113]. A study found that women with lower levels of magnesium and higher levels of Cu are more likely to suffer from depression [114]; while it is contradictory that the correlation between serum copper and the severity of depression was not found in another study [115].

Copper might influence depression via inflammation, oxidative stress, or synaptic plasticity. Copper exposure increases depression-like behavior and activates inflammation-related microglia in *APOE4* transgenic mice [116]. Melatonin (Mel) attenuates CU-induced oxidative stress and depression-like behavior by decreasing lipid peroxidation (LPO) and nitric oxide (NO) levels and enhancing superoxide dismutase (SOD) and catalase (CAT) activities in the rat hippocampus [117]. Liu et al. showed that copper levels are increased in the hippocampus of stressed mice, which can affect synaptic function by inhibiting the expression of GluN2B and PSD95 [118].

### 3.4. Iron(Fe)

Iron is an essential trace element for human growth and development and plays a key role in ensuring normal brain development and function [119]. Several studies have revealed that dietary iron deficiency increases the risk of depression [76,80,82,120,121]. In a case-control study, mothers with postpartum iron deficiency were shown to be three times more likely to develop postpartum depression [122]. Postpartum iron supplementation helps reduce postpartum depression [123]. Serum iron concentration was significantly decreased in many patients with major depression compared to healthy subjects (1.02 ± 0.02 vs. 1.30 ± 0.03 mg/L, *p* < 0.05 mg/L) [84]. A survey study found that people with a history of iron deficiency anemia have a higher risk of depression [124]. A study of type Ⅰ diabetes and depression found that patients with iron deficiency have a higher incidence of depression [125]. Not only does iron deficiency increase the incidence of depression, but iron excess is also associated with depression. A study indicated significant iron deposition in the thalamus of patients with depression [126].

The mechanism by which iron induces depression is unclear, although it may be partially related to the level of BDNF and oxidative stress. Brain-derived neurotrophic factor (BDNF) is widely expressed in developing and adult mammalian brains and is associated with development, neural regeneration, synaptic transmission, synaptic plasticity, and neurogenesis [31,127]. Ceruloplasmin is a ferroxidase involved in iron metabolism by converting Fe (2+) to Fe (3+). Texel et al. found that ceruloplasmin knock-out mice produce anxiety-like behaviors with significantly decreased levels of Fe and BDNF in the hippocampus [128]. Other studies have also shown that a low dose of iron is associated with low BDNF expression in the rat hippocampus [129,130]. A high dose of iron, possibly from iron accumulation, induces depressive-like behavior in rats [131]. Iron deposition is closely related to depression, and the possible mechanism is that iron deposition leads to increased production of reactive oxygen species, which in turn causes neuronal damage in the brain [132,133].

As Table 1 showed, we summarize the serum or blood Levels of elements in healthy subjects or major depressed patients and the possible mechanisms. 

## 4. Appropriate Supplementation of Some Mineral Elements or as Medication Can Help Alleviate Depression

### 4.1. Selenium(Se) Supplementation

Selenium is an essential trace element required for a variety of physiological functions, including thyroid hormone metabolism, protection against oxidative stress, and immune-related functions [134]. A cross-sectional study on US adults showed an inverse association between dietary selenium intake and depressive symptoms [76,135]. A survey on a Chinese rural elderly population [136] and a cross-sectional study on a Brazilian rural population [137] also indicated that higher selenium levels are associated with a lower prevalence of depression. The optimal doses for serum selenium in young Australians are between ∼82 μg/L and 85 μg/L, which results in a reduced risk of depressive symptoms [138]. Studies have shown that selenium supplementation may be an effective way to prevent postpartum depression [139,140]. However, a study on US youth found the opposite: higher selenium exposure levels are associated with increased depressive symptoms [141]. Therefore, selenium supplementation for depression requires the first measurement of selenium levels in depressed people, and further clinical studies are required. Selenium supplementation in rodents is beneficial for treating depression. The salt form of selenium, sodium selenite, had antidepressant effects in rodents [142]. Sodium selenite can increase the antidepressant effect of imipramine, fluoxetine, and tianeptine and reduce immobility time on the forced swim test (FST) after the administration of antidepressants [143].

The regulatory mechanisms of selenium in the treatment of depression are limited due to few experimental studies: 1. Anti-oxidative stress. Selenium can reverse arsenic-induced deterioration, alleviate depressive-like behaviors in the rat hippocampus, and reduce malondialdehyde levels and acetylcholinesterase activity [144]. Another study demonstrated that selenium could inhibit LPS-induced oxidative damage by increasing antioxidants [145]. 2. Anti-inflammatory. Fluoride treatment reduces dopamine and norepinephrine secretion, activates inflammation in microglia, and leads to depression-like behavior. While selenium treatment can activate the JAK2/STAT3 pathway, restore dopamine and norepinephrine secretion, reduce IL-1β secretion, and increase the number of viable cortical neurons, thus alleviating fluoride-induced depressive-like behavior [146].

### 4.2. Zinc(Zn) Supplementation

Section 2.1 of the article suggests that a zinc-deficient diet increases the risk of depression, and in fact, appropriate zinc supplementation can help treat depression. Clinical studies have shown that zinc supplementation, individually or in combination with antidepressants, may help reduce depressive symptoms, and the dose and treatment course of zinc in clinical trials were 25~220 mg for 6 to 12 weeks [147,148,149]. In chronic stress-induced rodent models of depression, zinc supplementation can reduce depressive-like behavior [150]. For depressed patients, zinc levels should be first measured. If zinc deficiency occurs, appropriate supplementation should be carried out, and zinc levels should be monitored during treatment.

The mechanisms of zinc treatment for depression are related to the levels of BDNF and anti-inflammation. A study showed that zinc enhances the antidepressant efficacy of imipramine by increasing the concentration of BDNF in the prefrontal cortex [151]. Kirsten et al. reported that zinc can inhibit LPS-induced depression-like behaviors caused by inflammation in rats and reduce the expression of inflammation-related factors IFN-γ [99].

### 4.3. Magnesium (Mg) Supplementation

Depressed patients with magnesium deficiency take 500 mg of magnesium oxide tablets daily for ≥8 weeks to alleviate depressive symptoms [152]. It is effective that over-the-counter magnesium chloride (248 mg of elemental magnesium per day) is taken by adults with mild to moderate symptoms of depression for 6 weeks [153]. A recent clinical study tracking the electroencephalogram in MDD patients showed that magnesium can enhance fluoxetine treatment in response to depression treatment [154]. As with the other elements above, an initial measurement of magnesium levels is required in depressed patients, followed by supplementing appropriately and monitoring magnesium levels during treatment.

The following are possible mechanisms of magnesium therapy for depression: 1. 5-HT. Poleszak et al. found that the antidepressant-like effects of magnesium are significantly reduced in mice pretreated with serotonin synthesis inhibitors [155]. 2. Anti-inflammation. Cyclophosphamide (CYP)-induced inflammation causes depression-like behaviors in rats and increases the inflammatory factors TNF-α and IL6, while supplementation with magnesium L-threonate could reduce the inflammatory response and depression-like behaviors [156]. 3. NMDA. Magnesium could treat depression-like behavior induced by chronic mild stress in rats, and concomitantly restore the levels of GluN1 and GluN2A and increase the levels of GluN2B and PSD-95 [157].

### 4.4. Lithium(Li) as a Psychotropic Medication

Lithium as a psychotropic medication has been used to treat bipolar disorder (BD) and prevent suicidal and depressive/manic episodes [158]. Lithium also plays an important role in the treatment of unipolar depression. Barroilhet et al. showed that a low dose of lithium in the body can help prevent suicide caused by depression, but a higher dose (over 1.0 mmol/L) has specific toxic side effects [159]. Adding trace amounts of lithium to drinking water may reduce suicide risk in the general population [160]. The addition of lithium to both imipramine and fluvoxamine in phase II is more effective for treating depression than the phase I drug alone [161]. Lithium has also been recommended in multiple depression medication guidelines [162]. Lithium is clinically effective as long-term monotherapy and supplemental antidepressant therapy for depression [163]. A cohort study in Finland reported that, compared to lithium combined with antidepressants, lithium alone has a lower risk for patients with significant depression readmitted to the hospital [164]. Other studies have also shown the benefits of lithium in treating depression [165,166]. Vázquez et al. summarized the dose and treatment course of lithium in clinical trials as 600~1200 mg for 1 to 6 weeks [167]. Since a higher dose (over 1.0 mmol/L) has specific toxic side effects, in the clinic, the lithium content should be first detected in depressed patients and then monitored regularly.

The possible physiological processes and mechanisms of lithium treatment for depression are considered as follows: 1. Hippocampal neurogenesis. Hippocampal adult neurogenesis is defined as new neurons generated in the dentate gyrus of the hippocampus and integrated into neural circuits during adulthood, which can repair nerve damage and increase neuroplasticity [168]. Hippocampal volume is reduced in the brains of depressed patients but increases after 3 years of antidepressant treatment, suggesting that antidepressants could induce hippocampal neurogenesis to alleviate depression [169]. In rodents, impaired neurogenesis can induce depression-like behaviors in animals [170]. An experimental study showed that both lithium alone and combined with fluoxetine could increase neurogenesis and eliminate depression-like behavior in resistant depression models. Moreover, lithium combined with fluoxetine has fewer side effects than lithium alone [171]. 2. BDNF. A survey has shown lithium can enhance serum BDNF levels in depressed patients [172]. Another study showed that lithium exerts an antidepressant effect by increasing BDNF and thereby increasing the firing activity of VTA-mPFC DA neurons in depressive-like mice [173]. 3. The blood–brain barrier (BBB). Disruption of the BBB leads to a disturbance of brain homeostasis that may be a key factor in the development of depression [174]. Lithium exerts antidepressant effects by protecting against the destruction of the BBB/neurovascular unit (NVU) in the chronic mild stress (CMS) rat model [175].

In Table 2, we summarize daily dose and course of mineral elements to treat depressed patients in clinical trials and the possible mechanisms.

## 5. Conclusions

**Figure 2:**
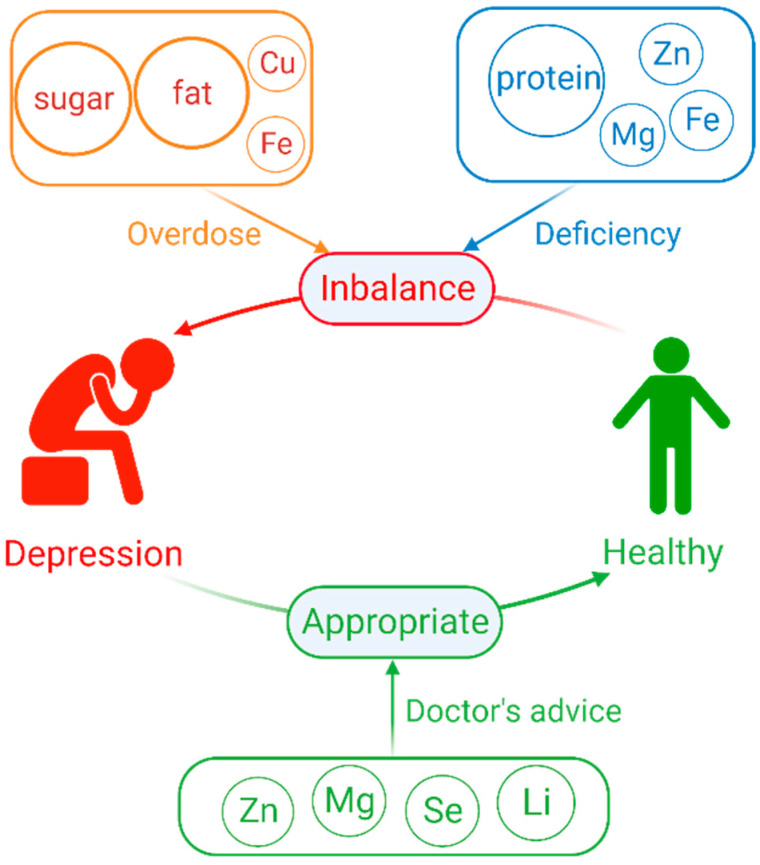
Summarize the relationship between depression and elements. Cu (copper), Fe (iron), Zn (zinc), Mg (magnesium), Se (selenium), Li (lithium).

Nutrients are indispensable to the human body and affect the normal physiological functions of the human body. As shown in Table 1, nutrient imbalances, including macronutrients (dietary sugars, fat, and protein) and mineral elements (zinc, magnesium, copper, and iron), may increase the occurrence of depression. Several clinical and non-clinical studies have shown that nutrients also affect the function of antidepressants. As summarized in Table 2, appropriate doses of selenium, zinc, magnesium, and lithium are beneficial for reducing depression. Magnesium and zinc deficiencies increase the risk of depression, and appropriate magnesium and zinc supplementation can also help improve depression. Therefore, as described in Figure 2, in addition to conventional drug treatment of depression, it also needs to pay attention to the level of the patient’s own nutritional elements, the timely supplementation of the lacking nutritional elements, the control of excessive nutritional elements, and the balance of these nutrients in the body. Moreover, nutritional elements such as lithium, selenium, zinc, magnesium, etc., for depression treatment should be based on elemental levels in the body and during treatment. Excessive elemental supplementation may be harmful; therefore, supplementation should be appropriate and follow the doctor’s advice and not be haphazard or uncontrolled.

In the serum or blood levels of depressed patients, fasting blood glucose, high-density lipoprotein-cholesterol, and copper are significantly elevated, while total protein, zinc, magnesium, and iron are significantly decreased [21,83,84], so further research should be conducted on whether these elements can be used as indicators for the prevention and treatment of depression.

This review summarizes the functions and mechanisms of some nutrients in depression research. Nevertheless, further studies are still required. For example, the intake of dietary sugar, fat, and protein in life also contains some additives such as sweeteners, preservatives, and other ingredients, and their relationships with depression are not clear, which should be addressed in follow-up studies. Furthermore, the nutritional elements associated with depression not only include those summarized in the article but also involve vitamins [176,177,178], folic acid [179], N-acetylcysteine [180], S-adenozylmetionine [181], dietary fiber [182], etc., which also need to be considered. This article suggests that to better prevent and treat depression, people should gradually focus on the role of nutrients in depression and their daily diet, such as low-sugar and low-fat diets.

In general, we summarized the involvement of some nutritional elements in depression and elucidated their related regulatory mechanisms. It may inspire novel preventive and therapeutic strategies for depression.

## Figures and Tables

**Table 1:** Summarize the possible mechanism for overdose or deficiency of macronutrients and mineral elements to increase the risk of depression.

Category		How to Increase the Risk of Depression	Serum or Blood Levels in Healthy Subjects	Serum or Blood Levels in Major Depressed Patients	Physiological Processes and Physiological Components
Macronutrients	Dietary sugars	Overdose	FBG: 4.52 ± 0.43 mmol/L	FBG:4.73 ± 0.45 mmol/L ** [21]	1. Neural signals: 5-HT↓ [22]
					2. Inflammation: pro-inflammatory factors such as IL-6, TNF-α, etc. ↑ [24]; gut microbiota [27]
					3. Synaptic plasticity: synapsin I and BDNF↓ [32]; Dendrite spines and dendritic branches↓ [33]
	Dietary fat	Overdose	TG: 1.08 [0.76–1.54] g/L	TG: 0.84 [0.63–1.32] g/L * [39]	1. Neural signals: 5-HT↓ [45]; 5-HT reabsorption↓ [46]; Intestinal 5-HT↑ [47]; GlutA2 and GAD65↓ [48]; desensitization of GABAergic AgRP neuron [49]; GLT-1↓ [50]
					2. Inflammation: pro-inflammatory factors such as IL-6, IL-1, TNF-α, etc. ↑ [45,46,51,52]
					3. Oxidative stress: TABRS, CAT, GPX↑ [51]
			HDL-C: 1.24 ± 0.30 mmol/L	HDL-C: 1.31 ± 0.32 mmol/L ** [21]	4. Synaptic plasticity: synapsin I and BDNF↓ [32]; βIII-tubulin, PSD-95, SNAP-25, and Neurotrophin-3↓ [48]
					5. Signaling pathway: Akt/GSK3β↓ [53]; cAMP/PKA↓ [54]; AMPK↓ [55].
					6. Other related receptor proteins: LepRb↓ [58], CNR1↓ [61]
	Dietary protein	Deficiency	TP: 68.72 ± 5.23 g/L	TP: 66.72 ± 5.10 g/L ** [21]	May be related to synthesis of 5-HT and dopamine [71,72]
Mineral elements	Zinc	Deficiency	0.96 ± 0.11 mg/L	0.72 ± 0.08 mg/L ** [83]	1. ZnT3↓ [89,91]; *ZnT3* knockout induced decreased hippocampal neurogenesis [92]
					2. GPR39 knockout [95]; GPR39 knockout induced decreased CREB and BDNF expression [96]
					3. Oxidation/inflammation parameters: IL-1 and TBARS↑ [98]
					4. Neural signals: NMDAR(GluN2A, GluN2B) ↑ [100,111]
	Magnesium	Deficiency	1.64 ± 0. 15 mg/L	1.10 ± 0.11 mg/L ** [83]	1. Gut microbiota [107]
					2. Neural signals: GluN1↓ [109]
					3. Oxidative stress: DDAH1, MnSOD, and GDH1↑ [110]; GPX↑ [111]
	Copper	Overdose	1.12 ± 0.13 mg/L	1.55 ± 0.12 mg/L ** [83]	1. Inflammation↑ [116]
					2. Oxidative stress: SOD and CAT↑ [117]
					3. Synaptic plasticity: GluN2B, PSD95↓ [118]
	Iron	Deficiency/overdose	1.30 ± 0.03 mg/L	1.02 ± 0.02 mg/L * [84]	May be related to BDNF↓ [129,130] and oxidative stress↑ [133]

Note: ↓: indicates lower or reduced; ↑: indicates increase or promotion. Depressed patients compared to healthy subjects, * *p* < 0.05; ** *p* < 0.01. Full name and abbreviation: fasting blood glucose (FBG), triglycerides (TG), high-density lipoprotein cholesterol (HDL-C), total protein (TP), 5-hydroxytryptamine (5-HT), interleukin-6 (IL-6), tumor necrosis factor-α (TNF-α), brain-derived neurotrophic factor (BDNF), glucose transporter A2 (GlutA2), glutamic acid decarboxylase 65-kilodalton isoform (GAD65), gamma-aminobutyric acid (GABA), agouti-related protein (AgRP), glutamate transporter 1 (GLT-1), interleukin-1 (IL-1), thiobarbituric acid reactive substances (TBARS), catalase (CAT), glutathione peroxidase (GPX), postsynaptic density protein 95 (PSD-95), synaptosomal-associated protein, 25 kDa (SNAP-25), protein kinase B (AKT), glycogen synthase kinase-3 (GSK3β), cyclic adenylic acid (cAMP), protein kinase A(PKA), adenosine 5′-monophosphate (AMP)-activated protein kinase (AMPK), leptin receptor (LepRb), cannabinoid receptor 1 (CNR1), zinc transporter 3 (ZnT3), G-protein coupled receptor 39 (GPR39), cAMP response element-binding protein (CREB), N-methyl-d-aspartate (NMDA) receptor 2A (GluN2A), N-methyl-d-aspartate receptor 2B (GluN2B), N-methyl-d-aspartate receptor 1 (GluN1), N(G), N(G)-dimethylarginine dimethylaminohydrolase 1 (DDAH1), manganese-superoxide dismutase (MnSOD), glutamate dehydrogenase 1 (GDH1), superoxide dismutase (SOD).

**Table 2:** A summary of the benefits of proper supplementation with some mineral elements or as medication that can help improve depression.

Category			Daily Dose and Course of Treatment in Depressed Patients in Clinical Trials	Physiological Processes and Physiological Components
Mineral elements	Proper supplementation	Selenium	—	1. Anti-oxidative stress [144,145]
				2. Anti-inflammatory: pro-inflammatory factors IL-1↓ [146]
		Zinc	25~220 mg for 8 to 12 weeks [147,148,149]	1. Anti-inflammatory: IFN-γ↓ [99]
				2. BDNF↑ [151]
		Magnesium	248~500 mg for 6 to 8 weeks [152,153]	1. 5-HT↑ [155]
				2. Anti-inflammation: TNF-α and IL6↓ [156]
				3. Glutamate signaling↑ [157]
	Psychotropic medication			1. Hippocampal neurogenesis↑ [171]
		Lithium	600~1200 mg for 1 to 6 weeks [167]	2. BDNF↑ [172,173]
				3. Protects the blood–brain barrier [175]

Note: ↓: indicates lower or reduced; ↑: indicates increase or promotion. —: not applicable. Full name and abbreviation: brain-derived neurotrophic factor (BDNF), interleukin-1 (IL-1), interferon-gamma (IFN-γ), 5-hydroxytryptamine (5-HT), tumor necrosis factor-α (TNF-α), interleukin-6 (IL-6).

## Data Availability

Not applicable.

## References

[1] World Health Organization Depression. http://www.who.int/mediacentre/factsheets/fs369/en/.

[2] Mrazek D.A., Hornberger J.C., Altar C.A., Degtiar I. (2014). A review of the clinical, economic, and societal burden of treatment-resistant depression: 1996–2013. Psychiatr. Serv..

[3] Hamon M., Blier P. (2013). Monoamine neurocircuitry in depression and strategies for new treatments. Prog. Neuro-Psychopharmacol. Biol. Psychiatry.

[4] Yu H., Chen Z.-Y. (2011). The role of BDNF in depression on the basis of its location in the neural circuitry. Acta Pharmacol. Sin..

[5] Beurel E., Toups M., Nemeroff C.B. (2020). The Bidirectional Relationship of Depression and Inflammation: Double Trouble. Neuron.

[6] Dwyer J.B., Aftab A., Radhakrishnan R., Widge A., Rodriguez C.I., Carpenter L.L., Nemeroff C.B., McDonald W.M., Kalin N.H. (2020). Hormonal Treatments for Major Depressive Disorder: State of the Art. Am. J. Psychiatry.

[7] Aly J., Engmann O. (2020). The Way to a Human’s Brain Goes Through Their Stomach: Dietary Factors in Major Depressive Disorder. Front. Neurosci..

[8] Shayganfard M. (2022). Are Essential Trace Elements Effective in Modulation of Mental Disorders? Update and Perspectives. Biol. Trace Elem. Res..

[9] Mergenthaler P., Lindauer U., Dienel G.A., Meisel A. (2013). Sugar for the brain: The role of glucose in physiological and pathological brain function. Trends Neurosci..

[10] Guo X., Park Y., Freedman N.D., Sinha R., Hollenbeck A.R., Blair A., Chen H. (2014). Sweetened beverages, coffee, and tea and depression risk among older US adults. PLoS ONE.

[11] Vermeulen E., Stronks K., Snijder M.B., Schene A.H., Lok A., de Vries J.H., Visser M., Brouwer I.A., Nicolaou M. (2017). A combined high-sugar and high-saturated-fat dietary pattern is associated with more depressive symptoms in a multi-ethnic population: The HELIUS (Healthy Life in an Urban Setting) study. Public Health Nutr..

[12] Shimmura N., Nanri A., Kashino I., Kochi T., Eguchi M., Kabe I., Mizoue T. (2022). Prospective association of confectionery intake with depressive symptoms among Japanese workers: The Furukawa Nutrition and Health Study. Br. J. Nutr..

[13] Kashino I., Kochi T., Imamura F., Eguchi M., Kuwahara K., Nanri A., Kurotani K., Akter S., Hu H., Miki T. (2021). Prospective association of soft drink consumption with depressive symptoms. Nutrition.

[14] Hu D., Cheng L., Jiang W. (2019). Sugar-sweetened beverages consumption and the risk of depression: A meta-analysis of observational studies. J. Affect. Disord..

[15] Yu B., He H., Zhang Q., Wu H., Du H., Liu L., Wang C., Shi H., Xia Y., Guo X. (2015). Soft drink consumption is associated with depressive symptoms among adults in China. J. Affect. Disord..

[16] Zhang X., Huang X., Xiao Y., Jing D., Huang Y., Chen L., Luo D., Chen X., Shen M. (2019). Daily intake of soft drinks is associated with symptoms of anxiety and depression in Chinese adolescents. Public Health Nutr..

[17] Sanchez-Villegas A., Zazpe I., Santiago S., Perez-Cornago A., Martinez-Gonzalez M.A., Lahortiga-Ramos F. (2018). Added sugars and sugar-sweetened beverage consumption, dietary carbohydrate index and depression risk in the Seguimiento Universidad de Navarra (SUN) Project. Br. J. Nutr..

[18] Kim J.-M., Lee E. (2021). Association between Soft-Drink Intake and Obesity, Depression, and Subjective Health Status of Male and Female Adults. Int. J. Environ. Res. Public Health.

[19] Pinna F., Suprani F., Deiana V., Lai L., Manchia M., Paribello P., Somaini G., Diana E., Nicotra E.F., Farci F. (2022). Depression in Diabetic Patients: What Is the Link With Eating Disorders? Results of a Study in a Representative Sample of Patients With Type 1 Diabetes. Front. Psychiatry.

[20] Borgland S.L. (2021). Can treatment of obesity reduce depression or vice versa?. J. Psychiatry Neurosci..

[21] Peng Y.-F., Xiang Y., Wei Y.-S. (2016). The significance of routine biochemical markers in patients with major depressive disorder. Sci. Rep..

[22] Inam Q.-u.-A., Jabeen B., Haleem M.A., Haleem D.J. (2008). Long-term consumption of sugar-rich diet decreases the effectiveness of somatodendritic serotonin-1A receptors. Nutr. Neurosci..

[23] Haase J., Brown E. (2015). Integrating the monoamine, neurotrophin and cytokine hypotheses of depression—A central role for the serotonin transporter?. Pharmacol. Ther..

[24] Köhler C.A., Freitas T.H., Maes M., de Andrade N.Q., Liu C.S., Fernandes B.S., Stubbs B., Solmi M., Veronese N., Herrmann N. (2017). Peripheral cytokine and chemokine alterations in depression: A meta-analysis of 82 studies. Acta Psychiatr. Scand..

[25] Sabedra Sousa F.S., Birmann P.T., Bampi S.R., Fronza M.G., Balaguez R., Alves D., Leite M.R., Nogueira C.W., Brüning C.A., Savegnago L. (2019). Lipopolysaccharide-induced depressive-like, anxiogenic-like and hyperalgesic behavior is attenuated by acute administration of α-(phenylselanyl) acetophenone in mice. Neuropharmacology.

[26] Casaril A.M., Domingues M., de Andrade Lourenço D., Birmann P.T., Padilha N., Vieira B., Begnini K., Seixas F.K., Collares T., Lenardão E.J. (2019). Depression- and anxiogenic-like behaviors induced by lipopolysaccharide in mice are reversed by a selenium-containing indolyl compound: Behavioral, neurochemical and computational insights involving the serotonergic system. J. Psychiatr. Res..

[27] Do M.H., Lee E., Oh M.-J., Kim Y., Park H.-Y. (2018). High-Glucose or -Fructose Diet Cause Changes of the Gut Microbiota and Metabolic Disorders in Mice without Body Weight Change. Nutrients.

[28] Sen S., Duman R., Sanacora G. (2008). Serum brain-derived neurotrophic factor, depression, and antidepressant medications: Meta-analyses and implications. Biol. Psychiatry.

[29] Kim Y.K., Lee H.P., Won S.D., Park E.Y., Lee H.Y., Lee B.H., Lee S.W., Yoon D., Han C., Kim D.J. (2007). Low plasma BDNF is associated with suicidal behavior in major depression. Prog. Neuropsychopharmacol. Biol. Psychiatry.

[30] Colucci-D’Amato L., Speranza L., Volpicelli F. (2020). Neurotrophic Factor BDNF, Physiological Functions and Therapeutic Potential in Depression, Neurodegeneration and Brain Cancer. Int. J. Mol. Sci..

[31] Björkholm C., Monteggia L.M. (2016). BDNF—A key transducer of antidepressant effects. Neuropharmacology.

[32] Molteni R., Barnard R.J., Ying Z., Roberts C.K., Gómez-Pinilla F. (2002). A high-fat, refined sugar diet reduces hippocampal brain-derived neurotrophic factor, neuronal plasticity, and learning. Neuroscience.

[33] Calvo-Ochoa E., Hernández-Ortega K., Ferrera P., Morimoto S., Arias C. (2014). Short-term high-fat-and-fructose feeding produces insulin signaling alterations accompanied by neurite and synaptic reduction and astroglial activation in the rat hippocampus. J. Cereb. Blood Flow Metab..

[34] Speed M.S., Jefsen O.H., Børglum A.D., Speed D., Østergaard S.D. (2019). Investigating the association between body fat and depression via Mendelian randomization. Transl. Psychiatry.

[35] Mannan M., Mamun A., Doi S., Clavarino A. (2016). Is there a bi-directional relationship between depression and obesity among adult men and women? Systematic review and bias-adjusted meta analysis. Asian J. Psychiatry.

[36] Mannan M., Mamun A., Doi S., Clavarino A. (2016). Prospective Associations between Depression and Obesity for Adolescent Males and Females—A Systematic Review and Meta-Analysis of Longitudinal Studies. PLoS ONE.

[37] Panth N., Dias C.B., Wynne K., Singh H., Garg M.L. (2020). Medium-chain fatty acids lower postprandial lipemia: A randomized crossover trial. Clin. Nutr..

[38] Oh J., Kim T.-S. (2017). Serum lipid levels in depression and suicidality: The Korea National Health and Nutrition Examination Survey (KNHANES) 2014. J. Affect. Disord..

[39] Enko D., Brandmayr W., Halwachs-Baumann G., Schnedl W.J., Meinitzer A., Kriegshäuser G. (2018). Prospective plasma lipid profiling in individuals with and without depression. Lipids Health Dis..

[40] So H.-C., Chau C.K.-L., Cheng Y.-Y., Sham P.C. (2021). Causal relationships between blood lipids and depression phenotypes: A Mendelian randomisation analysis. Psychol. Med..

[41] Braga S.P., Delanogare E., Machado A.E., Prediger R.D., Moreira E.L.G. (2021). Switching from high-fat feeding (HFD) to regular diet improves metabolic and behavioral impairments in middle-aged female mice. Behav. Brain Res..

[42] Yu H., Qin X., Yu Z., Chen Y., Tang L., Shan W. (2021). Effects of high-fat diet on the formation of depressive-like behavior in mice. Food Funct..

[43] Abildgaard A., Solskov L., Volke V., Harvey B.H., Lund S., Wegener G. (2011). A high-fat diet exacerbates depressive-like behavior in the Flinders Sensitive Line (FSL) rat, a genetic model of depression. Psychoneuroendocrinology.

[44] Mikami T., Kim J., Park J., Lee H., Yaicharoen P., Suidasari S., Yokozawa M., Yamauchi K. (2021). Olive leaf extract prevents obesity, cognitive decline, and depression and improves exercise capacity in mice. Sci. Rep..

[45] Wu H., Lv W., Pan Q., Kalavagunta P.K., Liu Q., Qin G., Cai M., Zhou L., Wang T., Xia Z. (2019). Simvastatin therapy in adolescent mice attenuates HFD-induced depression-like behavior by reducing hippocampal neuroinflammation. J. Affect. Disord..

[46] Hersey M., Woodruff J.L., Maxwell N., Sadek A.T., Bykalo M.K., Bain I., Grillo C.A., Piroli G.G., Hashemi P., Reagan L.P. (2021). High-fat diet induces neuroinflammation and reduces the serotonergic response to escitalopram in the hippocampus of obese rats. Brain Behav. Immun..

[47] Pan Q., Liu Q., Wan R., Kalavagunta P.K., Liu L., Lv W., Qiao T., Shang J., Wu H. (2019). Selective inhibition of intestinal 5-HT improves neurobehavioral abnormalities caused by high-fat diet mice. Metab. Brain Dis..

[48] Liu S., Xiu J., Zhu C., Meng K., Li C., Han R., Du T., Li L., Xu L., Liu R. (2021). Fat mass and obesity-associated protein regulates RNA methylation associated with depression-like behavior in mice. Nat. Commun..

[49] Xia G., Han Y., Meng F., He Y., Srisai D., Farias M., Dang M., Palmiter R.D., Xu Y., Wu Q. (2021). Reciprocal control of obesity and anxiety-depressive disorder via a GABA and serotonin neural circuit. Mol. Psychiatry.

[50] Tsai S.-F., Hsu P.-L., Chen Y.-W., Hossain M.S., Chen P.-C., Tzeng S.-F., Chen P.-S., Kuo Y.-M. (2022). High-fat diet induces depression-like phenotype via astrocyte-mediated hyperactivation of ventral hippocampal glutamatergic afferents to the nucleus accumbens. Mol. Psychiatry.

[51] Rebai R., Jasmin L., Boudah A. (2021). Agomelatine effects on fat-enriched diet induced neuroinflammation and depression-like behavior in rats. Biomed. Pharmacother. Biomed. Pharmacother..

[52] Wang H., Zhou J., Liu Q.Z., Wang L.L., Shang J. (2017). Simvastatin and Bezafibrate ameliorate Emotional disorder Induced by High fat diet in C57BL/6 mice. Sci. Rep..

[53] Arcego D.M., Toniazzo A.P., Krolow R., Lampert C., Berlitz C., Dos Santos Garcia E., do Couto Nicola F., Hoppe J.B., Gaelzer M.M., Klein C.P. (2018). Impact of High-Fat Diet and Early Stress on Depressive-Like Behavior and Hippocampal Plasticity in Adult Male Rats. Mol. Neurobiol..

[54] Vagena E., Ryu J.K., Baeza-Raja B., Walsh N.M., Syme C., Day J.P., Houslay M.D., Baillie G.S. (2019). A high-fat diet promotes depression-like behavior in mice by suppressing hypothalamic PKA signaling. Transl. Psychiatry.

[55] Li Y., Cheng Y., Zhou Y., Du H., Zhang C., Zhao Z., Chen Y., Zhou Z., Mei J., Wu W. (2022). High fat diet-induced obesity leads to depressive and anxiety-like behaviors in mice via AMPK/mTOR-mediated autophagy. Exp. Neurol..

[56] Liu W., Liu J., Xia J., Xue X., Wang H., Qi Z., Ji L. (2017). Leptin receptor knockout-induced depression-like behaviors and attenuated antidepressant effects of exercise are associated with STAT3/SOCS3 signaling. Brain Behav. Immun..

[57] Guo M., Huang T.-Y., Garza J.C., Chua S.C., Lu X.-Y. (2013). Selective deletion of leptin receptors in adult hippocampus induces depression-related behaviours. Int. J. Neuropsychopharmacol..

[58] Yang J.L., Liu D.X., Jiang H., Pan F., Ho C.S., Ho R.C. (2016). The Effects of High-fat-diet Combined with Chronic Unpredictable Mild Stress on Depression-like Behavior and Leptin/LepRb in Male Rats. Sci. Rep..

[59] Gallego-Landin I., García-Baos A., Castro-Zavala A., Valverde O. (2021). Reviewing the Role of the Endocannabinoid System in the Pathophysiology of Depression. Front. Pharm..

[60] Valverde O., Torrens M. (2012). CB1 receptor-deficient mice as a model for depression. Neuroscience.

[61] Gawliński D., Gawlińska K., Smaga I. (2021). Maternal High-Fat Diet Modulates Gene Expression in Male Rat Offspring. Nutrients.

[62] Oh J., Yun K., Chae J.-H., Kim T.-S. (2020). Association Between Macronutrients Intake and Depression in the United States and South Korea. Front. Psychiatry.

[63] Wolfe A.R., Arroyo C., Tedders S.H., Li Y., Dai Q., Zhang J. (2011). Dietary protein and protein-rich food in relation to severely depressed mood: A 10 year follow-up of a national cohort. Prog. Neuro-Psychopharmacol. Biol. Psychiatry.

[64] Li Y., Zhang C., Li S., Zhang D. (2020). Association between dietary protein intake and the risk of depressive symptoms in adults. Br. J. Nutr..

[65] Nanri A., Eguchi M., Kuwahara K., Kochi T., Kurotani K., Ito R., Pham N.M., Tsuruoka H., Akter S., Jacka F. (2014). Macronutrient intake and depressive symptoms among Japanese male workers: The Furukawa Nutrition and Health Study. Psychiatry Res..

[66] Nucci D., Fatigoni C., Amerio A., Odone A., Gianfredi V. (2020). Red and Processed Meat Consumption and Risk of Depression: A Systematic Review and Meta-Analysis. Int. J. Environ. Res. Public Health.

[67] Ciarambino T., Ferrara N., Castellino P., Paolisso G., Coppola L., Giordano M. (2011). Effects of a 6-days-a-week low protein diet regimen on depressive symptoms in young-old type 2 diabetic patients. Nutrition.

[68] Sun J., Wang W., Zhang D. (2020). Associations of different types of dairy intakes with depressive symptoms in adults. J. Affect. Disord..

[69] Badawy A.A. B. (2013). Tryptophan: The key to boosting brain serotonin synthesis in depressive illness. J. Psychopharmacol..

[70] Reuter M., Zamoscik V., Plieger T., Bravo R., Ugartemendia L., Rodriguez A.B., Kirsch P. (2021). Tryptophan-rich diet is negatively associated with depression and positively linked to social cognition. Nutr. Res..

[71] Franklin M., Bermudez I., Murck H., Singewald N., Gaburro S. (2012). Sub-chronic dietary tryptophan depletion—An animal model of depression with improved face and good construct validity. J. Psychiatr. Res..

[72] Papakostas G.I. (2006). Dopaminergic-based pharmacotherapies for depression. Eur. Neuropsychopharmacol..

[73] Vekovischeva O.Y., Peuhkuri K., Bäckström P., Sihvola N., Pilvi T., Korpela R. (2013). The effects of native whey and α-lactalbumin on the social and individual behaviour of C57BL/6J mice. Br. J. Nutr..

[74] Takeuchi T., Matsunaga K., Sugiyama A. (2017). Antidepressant-like effect of milk-derived lactoferrin in the repeated forced-swim stress mouse model. J. Vet. Med. Sci..

[75] Szewczyk B., Kubera M., Nowak G. (2011). The role of zinc in neurodegenerative inflammatory pathways in depression. Prog. Neuro-Psychopharmacol. Biol. Psychiatry.

[76] Li Z., Wang W., Xin X., Song X., Zhang D. (2018). Association of total zinc, iron, copper and selenium intakes with depression in the US adults. J. Affect. Disord..

[77] Vashum K.P., McEvoy M., Milton A.H., McElduff P., Hure A., Byles J., Attia J. (2014). Dietary zinc is associated with a lower incidence of depression: Findings from two Australian cohorts. J. Affect. Disord..

[78] Anbari-Nogyni Z., Bidaki R., Madadizadeh F., Sangsefidi Z.S., Fallahzadeh H., Karimi-Nazari E., Nadjarzadeh A. (2020). Relationship of zinc status with depression and anxiety among elderly population. Clin. Nutr. ESPEN.

[79] Nakamura M., Miura A., Nagahata T., Shibata Y., Okada E., Ojima T. (2019). Low Zinc, Copper, and Manganese Intake is Associated with Depression and Anxiety Symptoms in the Japanese Working Population: Findings from the Eating Habit and Well-Being Study. Nutrients.

[80] Miki T., Kochi T., Eguchi M., Kuwahara K., Tsuruoka H., Kurotani K., Ito R., Akter S., Kashino I., Pham N.M. (2015). Dietary intake of minerals in relation to depressive symptoms in Japanese employees: The Furukawa Nutrition and Health Study. Nutrition.

[81] Maserejian N.N., Hall S.A., McKinlay J.B. (2012). Low dietary or supplemental zinc is associated with depression symptoms among women, but not men, in a population-based epidemiological survey. J. Affect. Disord..

[82] Thi Thu Nguyen T., Miyagi S., Tsujiguchi H., Kambayashi Y., Hara A., Nakamura H., Suzuki K., Yamada Y., Shimizu Y., Nakamura H. (2019). Association between Lower Intake of Minerals and Depressive Symptoms among Elderly Japanese Women but Not Men: Findings from Shika Study. Nutrients.

[83] Al-Fartusie F.S., Al-Bairmani H.K., Al-Garawi Z.S., Yousif A.H. (2019). Evaluation of Some Trace Elements and Vitamins in Major Depressive Disorder Patients: A Case-Control Study. Biol. Trace Elem. Res..

[84] Islam M.R., Islam M.R., Shalahuddin Qusar M.M.A., Islam M.S., Kabir M.H., Mustafizur Rahman G.K.M., Islam M.S., Hasnat A. (2018). Alterations of serum macro-minerals and trace elements are associated with major depressive disorder: A case-control study. BMC Psychiatry.

[85] Whittle N., Lubec G., Singewald N. (2009). Zinc deficiency induces enhanced depression-like behaviour and altered limbic activation reversed by antidepressant treatment in mice. Amino Acids.

[86] Tassabehji N.M., Corniola R.S., Alshingiti A., Levenson C.W. (2008). Zinc deficiency induces depression-like symptoms in adult rats. Physiol. Behav..

[87] Młyniec K., Nowak G. (2012). Zinc deficiency induces behavioral alterations in the tail suspension test in mice. Effect of antidepressants. Pharmacol. Rep. PR.

[88] Thingholm T.E., Rönnstrand L., Rosenberg P.A. (2020). Why and how to investigate the role of protein phosphorylation in ZIP and ZnT zinc transporter activity and regulation. Cell Mol. Life Sci..

[89] Rafalo-Ulinska A., Piotrowska J., Kryczyk A., Opoka W., Sowa-Kucma M., Misztak P., Rajkowska G., Stockmeier C.A., Datka W., Nowak G. (2016). Zinc transporters protein level in postmortem brain of depressed subjects and suicide victims. J. Psychiatr. Res..

[90] McAllister B.B., Dyck R.H. (2017). Zinc transporter 3 (ZnT3) and vesicular zinc in central nervous system function. Neurosci. Biobehav. Rev..

[91] Dou X., Tian X., Zheng Y., Huang J., Shen Z., Li H., Wang X., Mo F., Wang W., Wang S. (2014). Psychological stress induced hippocampus zinc dyshomeostasis and depression-like behavior in rats. Behav. Brain Res..

[92] Suh S.W., Won S.J., Hamby A.M., Yoo B.H., Fan Y., Sheline C.T., Tamano H., Takeda A., Liu J. (2009). Decreased brain zinc availability reduces hippocampal neurogenesis in mice and rats. J. Cereb. Blood Flow Metab..

[93] Laitakari A., Liu L., Frimurer T.M., Holst B. (2021). The Zinc-Sensing Receptor GPR39 in Physiology and as a Pharmacological Target. Int. J. Mol. Sci..

[94] Siodłak D., Nowak G., Mlyniec K. (2021). Interaction between zinc, the GPR39 zinc receptor and the serotonergic system in depression. Brain Res. Bull..

[95] Młyniec K., Gaweł M., Nowak G. (2015). Study of antidepressant drugs in GPR39 (zinc receptor^−/−^) knockout mice, showing no effect of conventional antidepressants, but effectiveness of NMDA antagonists. Behav. Brain Res..

[96] Młyniec K., Budziszewska B., Holst B., Ostachowicz B., Nowak G. (2014). GPR39 (zinc receptor) knockout mice exhibit depression-like behavior and CREB/BDNF down-regulation in the hippocampus. Int. J. Neuropsychopharmacol..

[97] Mlyniec K. (2021). Interaction between Zinc, GPR39, BDNF and Neuropeptides in Depression. Curr. Neuropharmacol..

[98] Doboszewska U., Szewczyk B., Sowa-Kućma M., Noworyta-Sokołowska K., Misztak P., Gołębiowska J., Młyniec K., Ostachowicz B., Krośniak M., Wojtanowska-Krośniak A. (2016). Alterations of Bio-elements, Oxidative, and Inflammatory Status in the Zinc Deficiency Model in Rats. Neurotox. Res..

[99] Kirsten T.B., Cabral D., Galvão M.C., Monteiro R., Bondan E.F., Bernardi M.M. (2020). Zinc, but not paracetamol, prevents depressive-like behavior and sickness behavior, and inhibits interferon-gamma and astrogliosis in rats. Brain Behav. Immun..

[100] Doboszewska U., Szewczyk B., Sowa-Kućma M., Młyniec K., Rafało A., Ostachowicz B., Lankosz M., Nowak G. (2015). Antidepressant activity of fluoxetine in the zinc deficiency model in rats involves the NMDA receptor complex. Behav. Brain Res..

[101] Doboszewska U., Sowa-Kućma M., Młyniec K., Pochwat B., Hołuj M., Ostachowicz B., Pilc A., Nowak G., Szewczyk B. (2015). Zinc deficiency in rats is associated with up-regulation of hippocampal NMDA receptor. Prog. Neuro-Psychopharmacol. Biol. Psychiatry.

[102] Botturi A., Ciappolino V., Delvecchio G., Boscutti A., Viscardi B., Brambilla P. (2020). The Role and the Effect of Magnesium in Mental Disorders: A Systematic Review. Nutrients.

[103] Tarleton E.K., Kennedy A.G., Rose G.L., Crocker A., Littenberg B. (2019). The Association between Serum Magnesium Levels and Depression in an Adult Primary Care Population. Nutrients.

[104] Sun C., Wang R., Li Z., Zhang D. (2019). Dietary magnesium intake and risk of depression. J. Affect. Disord..

[105] Li B., Lv J., Wang W., Zhang D. (2017). Dietary magnesium and calcium intake and risk of depression in the general population: A meta-analysis. Aust. N. Z. J. Psychiatry.

[106] Singewald N., Sinner C., Hetzenauer A., Sartori S.B., Murck H. (2004). Magnesium-deficient diet alters depression- and anxiety-related behavior in mice—Influence of desipramine and Hypericum perforatum extract. Neuropharmacology.

[107] Winther G., Pyndt Jørgensen B.M., Elfving B., Nielsen D.S., Kihl P., Lund S., Sørensen D.B., Wegener G. (2015). Dietary magnesium deficiency alters gut microbiota and leads to depressive-like behaviour. Acta Neuropsychiatr..

[108] Del Chierico F., Trapani V., Petito V., Reddel S., Pietropaolo G., Graziani C., Masi L., Gasbarrini A., Putignani L., Scaldaferri F. (2021). Dietary Magnesium Alleviates Experimental Murine Colitis through Modulation of Gut Microbiota. Nutrients.

[109] Ghafari M., Whittle N., Miklósi A.G., Kotlowski C., Kotlowsky C., Schmuckermair C., Berger J., Bennett K.L., Singewald N., Lubec G. (2015). Dietary magnesium restriction reduces amygdala-hypothalamic GluN1 receptor complex levels in mice. Brain Struct. Funct..

[110] Whittle N., Li L., Chen W.-Q., Yang J.-W., Sartori S.B., Lubec G., Singewald N. (2011). Changes in brain protein expression are linked to magnesium restriction-induced depression-like behavior. Amino Acids.

[111] Opanković A., Milovanović S., Radosavljević B., Čavić M., Besu Žižak I., Bukumirić Z., Latas M., Medić B., Vučković S., Srebro D. (2022). Correlation of Ionized Magnesium with the Parameters of Oxidative Stress as Potential Biomarkers in Patients with Anxiety and Depression: A Pilot Study. Dose Response.

[112] Scheiber I.F., Mercer J.F.B., Dringen R. (2014). Metabolism and functions of copper in brain. Prog. Neurobiol..

[113] Ni M., You Y., Chen J., Zhang L. (2018). Copper in depressive disorder: A systematic review and meta-analysis of observational studies. Psychiatry Res..

[114] Szkup M., Jurczak A., Brodowska A., Brodowska A., Noceń I., Chlubek D., Laszczyńska M., Karakiewicz B., Grochans E. (2017). Analysis of Relations Between the Level of Mg, Zn, Ca, Cu, and Fe and Depressiveness in Postmenopausal Women. Biol. Trace Elem. Res..

[115] Styczeń K., Sowa-Kućma M., Siwek M., Dudek D., Reczyński W., Misztak P., Szewczyk B., Topór-Mądry R., Opoka W., Nowak G. (2016). Study of the Serum Copper Levels in Patients with Major Depressive Disorder. Biol. Trace Elem. Res..

[116] Xu J., He K., Zhang K., Yang C., Nie L., Dan D., Liu J., Zhang C.-E., Yang X. (2021). Low-Dose Copper Exposure Exacerbates Depression-Like Behavior in ApoE4 Transgenic Mice. Oxidative Med. Cell. Longev..

[117] Lamtai M., Zghari O., Azirar S., Ouakki S., Mesfioui A., El Hessni A., Berkiks I., Marmouzi I., Ouichou A. (2021). Melatonin modulates copper-induced anxiety-like, depression-like and memory impairments by acting on hippocampal oxidative stress in rat. Drug Chem. Toxicol..

[118] Liu X., Lin C., Wang S., Yu X., Jia Y., Chen J. (2022). Effects of high levels of copper on the depression-related memory disorders. J. Gerontol. A Biol. Sci. Med. Sci..

[119] Barks A., Hall A.M., Tran P.V., Georgieff M.K. (2019). Iron as a model nutrient for understanding the nutritional origins of neuropsychiatric disease. Pediatr. Res..

[120] Li Z., Li B., Song X., Zhang D. (2017). Dietary zinc and iron intake and risk of depression: A meta-analysis. Psychiatry Res..

[121] Portugal-Nunes C., Castanho T.C., Amorim L., Moreira P.S., Mariz J., Marques F., Sousa N., Santos N.C., Palha J.A. (2020). Iron Status is Associated with Mood, Cognition, and Functional Ability in Older Adults: A Cross-Sectional Study. Nutrients.

[122] Hameed S., Naser I.A., Al Ghussein M.A., Ellulu M.S. (2021). Is iron deficiency a risk factor for postpartum depression? A case-control study in the Gaza Strip, Palestine. Public Health Nutr..

[123] Tian Y., Zheng Z., Ma C. (2020). The effectiveness of iron supplementation for postpartum depression: A protocol for systematic review and meta-analysis. Medicine.

[124] Hidese S., Saito K., Asano S., Kunugi H. (2018). Association between iron-deficiency anemia and depression: A web-based Japanese investigation. Psychiatry Clin. Neurosci..

[125] Bergis D., Tessmer L., Badenhoop K. (2019). Iron deficiency in long standing type 1 diabetes mellitus and its association with depression and impaired quality of life. Diabetes Res. Clin. Pract..

[126] Zhang W., Zhou Y., Li Q., Xu J., Yan S., Cai J., Jiaerken Y., Lou M. (2019). Brain Iron Deposits in Thalamus Is an Independent Factor for Depressive Symptoms Based on Quantitative Susceptibility Mapping in an Older Adults Community Population. Front. Psychiatry.

[127] Autry A.E., Monteggia L.M. (2012). Brain-derived neurotrophic factor and neuropsychiatric disorders. Pharm. Rev..

[128] Texel S.J., Camandola S., Ladenheim B., Rothman S.M., Mughal M.R., Unger E.L., Cadet J.L., Mattson M.P. (2012). Ceruloplasmin deficiency results in an anxiety phenotype involving deficits in hippocampal iron, serotonin, and BDNF. J. Neurochem..

[129] Tran P.V., Carlson E.S., Fretham S.J.B., Georgieff M.K. (2008). Early-life iron deficiency anemia alters neurotrophic factor expression and hippocampal neuron differentiation in male rats. J. Nutr..

[130] Tran P.V., Fretham S.J.B., Carlson E.S., Georgieff M.K. (2009). Long-term reduction of hippocampal brain-derived neurotrophic factor activity after fetal-neonatal iron deficiency in adult rats. Pediatr. Res..

[131] Mehrpouya S., Nahavandi A., Khojasteh F., Soleimani M., Ahmadi M., Barati M. (2015). Iron administration prevents BDNF decrease and depressive-like behavior following chronic stress. Brain Res..

[132] Wang F., Zhang M., Li Y., Li Y., Gong H., Li J., Zhang Y., Zhang C., Yan F., Sun B. (2022). Alterations in brain iron deposition with progression of late-life depression measured by magnetic resonance imaging (MRI)-based quantitative susceptibility mapping. Quant. Imaging Med. Surg..

[133] Youdim M.B. H. (2018). Monoamine oxidase inhibitors, and iron chelators in depressive illness and neurodegenerative diseases. J. Neural Transm..

[134] Baldessarini R.J., Tondo L., Vázquez G.H. (2019). Pharmacological treatment of adult bipolar disorder. Mol. Psychiatry.

[135] Barroilhet S.A., Ghaemi S.N. (2020). When and how to use lithium. Acta Psychiatr. Scand..

[136] Memon A., Rogers I., Fitzsimmons S.M.D.D., Carter B., Strawbridge R., Hidalgo-Mazzei D., Young A.H. (2020). Association between naturally occurring lithium in drinking water and suicide rates: Systematic review and meta-analysis of ecological studies. Br. J. Psychiatry J. Ment. Sci..

[137] Birkenhäger T.K., van den Broek W.W., Mulder P.G., Bruijn J.A., Moleman P. (2004). Comparison of two-phase treatment with imipramine or fluvoxamine, both followed by lithium addition, in inpatients with major depressive disorder. Am. J. Psychiatry.

[138] Taylor R.W., Marwood L., Oprea E., DeAngel V., Mather S., Valentini B., Zahn R., Young A.H., Cleare A.J. (2020). Pharmacological Augmentation in Unipolar Depression: A Guide to the Guidelines. Int. J. Neuropsychopharmacol..

[139] Undurraga J., Sim K., Tondo L., Gorodischer A., Azua E., Tay K.H., Tan D., Baldessarini R.J. (2019). Lithium treatment for unipolar major depressive disorder: Systematic review. J. Psychopharmacol..

[140] Tiihonen J., Tanskanen A., Hoti F., Vattulainen P., Taipale H., Mehtälä J., Lähteenvuo M. (2017). Pharmacological treatments and risk of readmission to hospital for unipolar depression in Finland: A nationwide cohort study. Lancet Psychiatry.

[141] Maruki T., Utsumi T., Takeshima M., Fujiwara Y., Matsui M., Aoki Y., Toda H., Watanabe N., Watanabe K., Takaesu Y. (2022). Efficacy and safety of adjunctive therapy to lamotrigine, lithium, or valproate monotherapy in bipolar depression: A systematic review and meta-analysis of randomized controlled trials. Int. J. Bipolar Disord..

[142] Rakofsky J.J., Lucido M.J., Dunlop B.W. (2022). Lithium in the treatment of acute bipolar depression: A systematic review and meta-analysis. J. Affect. Disord..

[143] Vázquez G.H., Bahji A., Undurraga J., Tondo L., Baldessarini R.J. (2021). Efficacy and Tolerability of Combination Treatments for Major Depression: Antidepressants plus Second-Generation Antipsychotics vs. Esketamine vs. Lithium. J. Psychopharmacol..

[144] Denoth-Lippuner A., Jessberger S. (2021). Formation and integration of new neurons in the adult hippocampus. Nat. Rev. Neurosci..

[145] Frodl T., Jäger M., Smajstrlova I., Born C., Bottlender R., Palladino T., Reiser M., Möller H.-J., Meisenzahl E.M. (2008). Effect of hippocampal and amygdala volumes on clinical outcomes in major depression: A 3-year prospective magnetic resonance imaging study. J. Psychiatry Neurosci..

[146] Egeland M., Guinaudie C., Du Preez A., Musaelyan K., Zunszain P.A., Fernandes C., Pariante C.M., Thuret S. (2017). Depletion of adult neurogenesis using the chemotherapy drug temozolomide in mice induces behavioural and biological changes relevant to depression. Transl. Psychiatry.

[147] Kin K., Yasuhara T., Kawauchi S., Kameda M., Hosomoto K., Tomita Y., Umakoshi M., Kuwahara K., Kin I., Kidani N. (2019). Lithium counteracts depressive behavior and augments the treatment effect of selective serotonin reuptake inhibitor in treatment-resistant depressed rats. Brain Res..

[148] Ricken R., Adli M., Lange C., Krusche E., Stamm T.J., Gaus S., Koehler S., Nase S., Bschor T., Richter C. (2013). Brain-derived neurotrophic factor serum concentrations in acute depressive patients increase during lithium augmentation of antidepressants. J. Clin. Psychopharmacol..

[149] Liu D., Tang Q.-Q., Wang D., Song S.-P., Yang X.-N., Hu S.-W., Wang Z.-Y., Xu Z., Liu H., Yang J.-X. (2020). Mesocortical BDNF signaling mediates antidepressive-like effects of lithium. Neuropsychopharmacol. Off. Publ. Am. Coll. Neuropsychopharmacol..

[150] Wu S., Yin Y., Du L. (2021). Blood-Brain Barrier Dysfunction in the Pathogenesis of Major Depressive Disorder. Cell. Mol. Neurobiol..

[151] Taler M., Aronovich R., Henry Hornfeld S., Dar S., Sasson E., Weizman A., Hochman E. (2021). Regulatory effect of lithium on hippocampal blood-brain barrier integrity in a rat model of depressive-like behavior. Bipolar Disord..

[152] Barchielli G., Capperucci A., Tanini D. (2022). The Role of Selenium in Pathologies: An Updated Review. Antioxidants.

[153] Ghimire S., Baral B.K., Feng D., Sy F.S., Rodriguez R. (2019). Is selenium intake associated with the presence of depressive symptoms among US adults? Findings from National Health and Nutrition Examination Survey (NHANES) 2011–2014. Nutrition.

[154] Gao S., Jin Y., Unverzagt F.W., Liang C., Hall K.S., Cao J., Ma F., Murrell J.R., Cheng Y., Li P. (2012). Selenium level and depressive symptoms in a rural elderly Chinese cohort. BMC Psychiatry.

[155] Ferreira de Almeida T.L., Petarli G.B., Cattafesta M., Zandonade E., Bezerra O.M.d.P.A., Tristão K.G., Salaroli L.B. (2021). Association of Selenium Intake and Development of Depression in Brazilian Farmers. Front. Nutr..

[156] Conner T.S., Richardson A.C., Miller J.C. (2015). Optimal serum selenium concentrations are associated with lower depressive symptoms and negative mood among young adults. J. Nutr..

[157] Leung B.M.Y., Kaplan B.J., Field C.J., Tough S., Eliasziw M., Gomez M.F., McCargar L.J., Gagnon L. (2013). Prenatal micronutrient supplementation and postpartum depressive symptoms in a pregnancy cohort. BMC Pregnancy Childbirth.

[158] Mokhber N., Namjoo M., Tara F., Boskabadi H., Rayman M.P., Ghayour-Mobarhan M., Sahebkar A., Majdi M.R., Tavallaie S., Azimi-Nezhad M. (2011). Effect of supplementation with selenium on postpartum depression: A randomized double-blind placebo-controlled trial. J. Matern. Fetal Neonatal Med. Off. J. Eur. Assoc. Perinat. Med. Fed. Asia Ocean. Perinat. Soc. Int. Soc. Perinat. Obstet..

[159] Colangelo L.A., He K., Whooley M.A., Daviglus M.L., Morris S., Liu K. (2014). Selenium exposure and depressive symptoms: The Coronary Artery Risk Development in Young Adults Trace Element Study. Neurotoxicology.

[160] Kędzierska E., Dudka J., Poleszak E., Kotlińska J.H. (2017). Antidepressant and anxiolytic-like activity of sodium selenite after acute treatment in mice. Pharmacol. Rep. PR.

[161] Kędzierska E., Dąbkowska L., Obierzyński P., Polakowska M., Poleszak E., Wlaź P., Szewczyk K., Kotlińska J. (2018). Synergistic Action of Sodium Selenite with some Antidepressants and Diazepam in Mice. Pharmaceutics.

[162] Samad N., Rao T., Rehman M.H.U., Bhatti S.A., Imran I. (2022). Inhibitory Effects of Selenium on Arsenic-Induced Anxiety-/Depression-Like Behavior and Memory Impairment. Biol. Trace Elem. Res..

[163] Zhang B., Guo Y., Yan S., Guo X., Zhao Y., Shi B. (2020). The protective effect of selenium on the lipopolysaccharide-induced oxidative stress and depressed gene expression related to milk protein synthesis in bovine mammary epithelial cells. Biol. Trace Elem. Res..

[164] Yang J., Li H., Hao Z., Jing X., Zhao Y., Cheng X., Ma H., Wang J., Wang J. (2022). Mitigation Effects of Selenium Nanoparticles on Depression-Like Behavior Induced by Fluoride in Mice via the JAK2-STAT3 Pathway. ACS Appl. Mater. Interfaces.

[165] Yosaee S., Clark C.C.T., Keshtkaran Z., Ashourpour M., Keshani P., Soltani S. (2022). Zinc in depression: From development to treatment: A comparative/dose response meta-analysis of observational studies and randomized controlled trials. Gen. Hosp. Psychiatry.

[166] Ranjbar E., Shams J., Sabetkasaei M., M-Shirazi M., Rashidkhani B., Mostafavi A., Bornak E., Nasrollahzadeh J. (2014). Effects of zinc supplementation on efficacy of antidepressant therapy, inflammatory cytokines, and brain-derived neurotrophic factor in patients with major depression. Nutr. Neurosci..

[167] Donig A., Hautzinger M. (2022). Zinc as an adjunct to antidepressant medication: A meta-analysis with subgroup analysis for different levels of treatment response to antidepressants. Nutr. Neurosci..

[168] Misztak P., Sowa-Kućma M., Pańczyszyn-Trzewik P., Szewczyk B., Nowak G. (2021). Antidepressant-like Effects of Combined Fluoxetine and Zinc Treatment in Mice Exposed to Chronic Restraint Stress Are Related to Modulation of Histone Deacetylase. Molecules.

[169] Rafało-Ulińska A., Poleszak E., Szopa A., Serefko A., Rogowska M., Sowa I., Wójciak M., Muszyńska B., Krakowska A., Gdula-Argasińska J. (2020). Imipramine Influences Body Distribution of Supplemental Zinc Which May Enhance Antidepressant Action. Nutrients.

[170] Rajizadeh A., Mozaffari-Khosravi H., Yassini-Ardakani M., Dehghani A. (2017). Effect of magnesium supplementation on depression status in depressed patients with magnesium deficiency: A randomized, double-blind, placebo-controlled trial. Nutrition.

[171] Tarleton E.K., Littenberg B., MacLean C.D., Kennedy A.G., Daley C. (2017). Role of magnesium supplementation in the treatment of depression: A randomized clinical trial. PLoS ONE.

[172] Skalski M., Mach A., Januszko P., Ryszewska-Pokraśniewicz B., Biernacka A., Nowak G., Pilc A., Poleszak E., Radziwoń-Zaleska M. (2021). Pharmaco-Electroencephalography-Based Assessment of Antidepressant Drug Efficacy-The Use of Magnesium Ions in the Treatment of Depression. J. Clin. Med..

[173] Poleszak E. (2007). Modulation of antidepressant-like activity of magnesium by serotonergic system. J. Neural Transm..

[174] Chen J.-L., Zhou X., Liu B.-L., Wei X.-H., Ding H.-L., Lin Z.-J., Zhan H.-L., Yang F., Li W.-B., Xie J.-C. (2020). Normalization of magnesium deficiency attenuated mechanical allodynia, depressive-like behaviors, and memory deficits associated with cyclophosphamide-induced cystitis by inhibiting TNF-α/NF-κB signaling in female rats. J. Neuroinflamm..

[175] Pochwat B., Szewczyk B., Sowa-Kucma M., Siwek A., Doboszewska U., Piekoszewski W., Gruca P., Papp M., Nowak G. (2014). Antidepressant-like activity of magnesium in the chronic mild stress model in rats: Alterations in the NMDA receptor subunits. Int. J. Neuropsychopharmacol..

[176] Ronaldson A., Arias de la Torre J., Gaughran F., Bakolis I., Hatch S.L., Hotopf M., Dregan A. (2022). Prospective associations between vitamin D and depression in middle-aged adults: Findings from the UK Biobank cohort. Psychol. Med..

[177] Ding J., Zhang Y. (2022). Associations of Dietary Vitamin C and E Intake With Depression. A Meta-Analysis of Observational Studies. Front. Nutr..

[178] Wu Y., Zhang L., Li S., Zhang D. (2022). Associations of dietary vitamin B1, vitamin B2, vitamin B6, and vitamin B12 with the risk of depression: A systematic review and meta-analysis. Nutr. Rev..

[179] Lam N.S.K., Long X.X., Li X., Saad M., Lim F., Doery J.C., Griffin R.C., Galletly C. (2022). The potential use of folate and its derivatives in treating psychiatric disorders: A systematic review. Biomed. Pharmacother..

[180] Smaga I., Frankowska M., Filip M. (2021). N-acetylcysteine as a new prominent approach for treating psychiatric disorders. Br. J. Pharmacol..

[181] Ullah H., Di Minno A., Esposito C., El-Seedi H.R., Khalifa S.A.M., Baldi A., Greco A., Santonastaso S., Cioffi V., Sperandeo R. (2022). Efficacy of a food supplement based on S-adenosyl methionine and probiotic strains in subjects with subthreshold depression and mild-to-moderate depression: A monocentric, randomized, cross-over, double-blind, placebo-controlled clinical trial. Biomed. Pharmacother..

[182] Xia Y., Liu Y., Zhang S., Zhang Q., Liu L., Meng G., Wu H., Sun S., Wang X., Zhou M. (2021). Associations between different types and sources of dietary fibre intake and depressive symptoms in a general population of adults: A cross-sectional study. Br. J. Nutr..

